# Supplementary material for: Cryo-EM structures of ρ1 GABAA receptors with antagonist and agonist drugs
Source: Nat Commun. 2025 Aug 1;16:7077. doi: 10.1038/s41467-025-61932-6 (PMC12316911; doi:10.1038/s41467-025-61932-6)
Supplement: Supplementary file 1 — Supplementary Information [file 41467_2025_61932_MOESM1_ESM.pdf]

Cryo-EM structures of ρ1 $GABA_A$ receptors with antagonist and agonist drugs

SUPPLEMENTARY INFORMATION

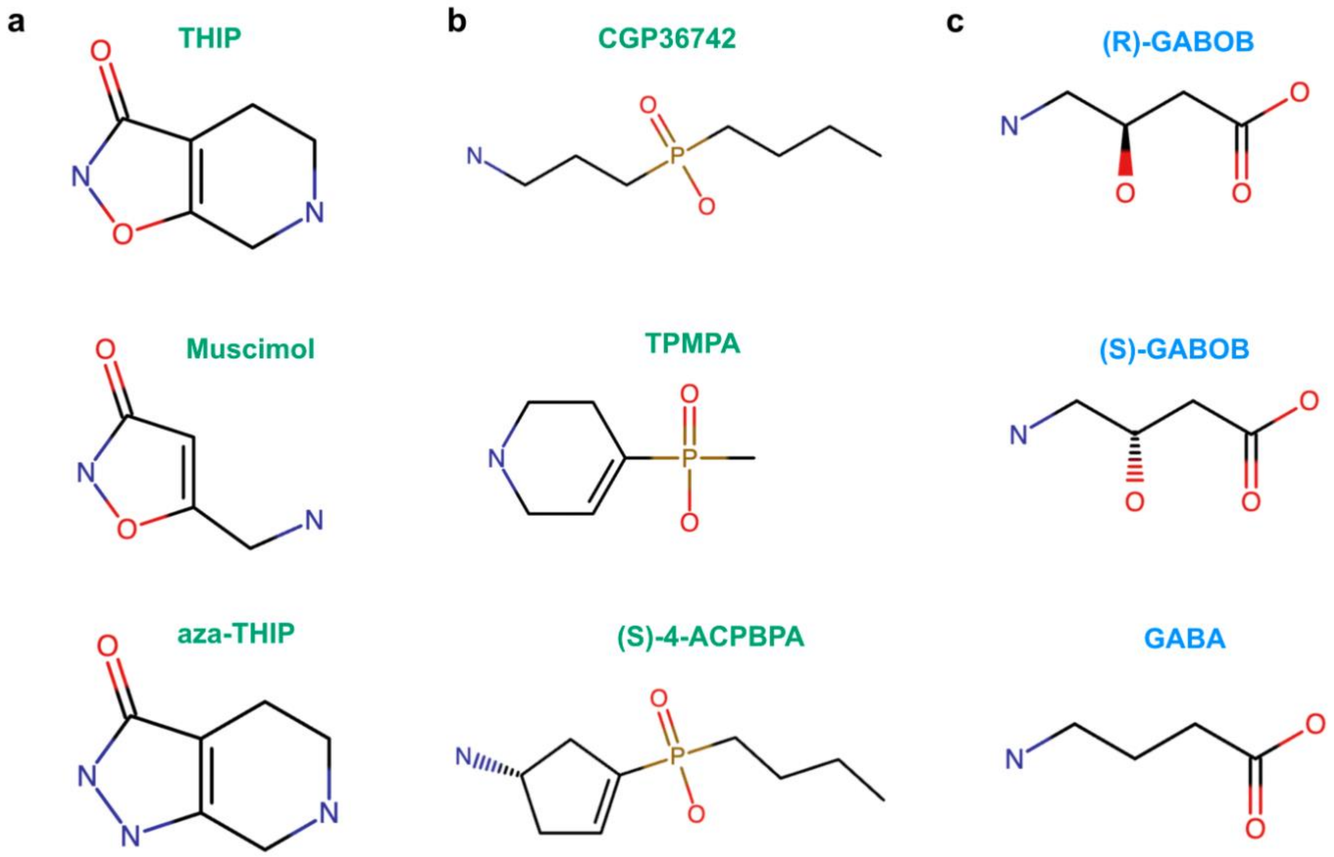

Supplementary Fig. 1. Chemical structures of relevant compounds.
(a) Chemical structures of THIP, muscimol and aza-THIP.
(b) Chemical structures of CGP36742 and TPMPA and (S)-4-ACPBPA.
(c) Chemical structures of (R)- and (S)-GABOB and GABA.

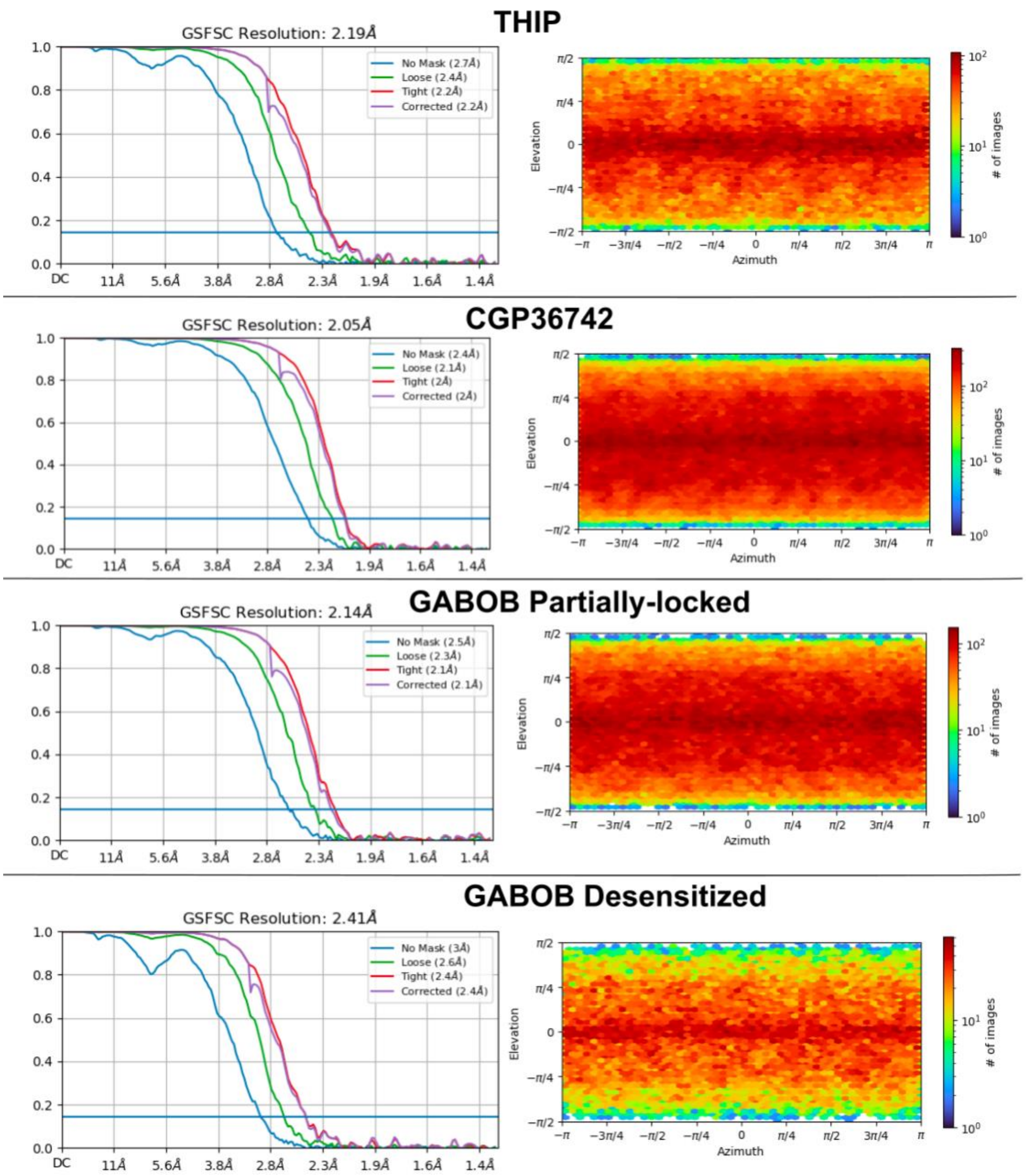

Supplementary Fig. 2. Fourier shell correlation (FSC) curves (left) and angular distributions (right) of cryo-EM maps.

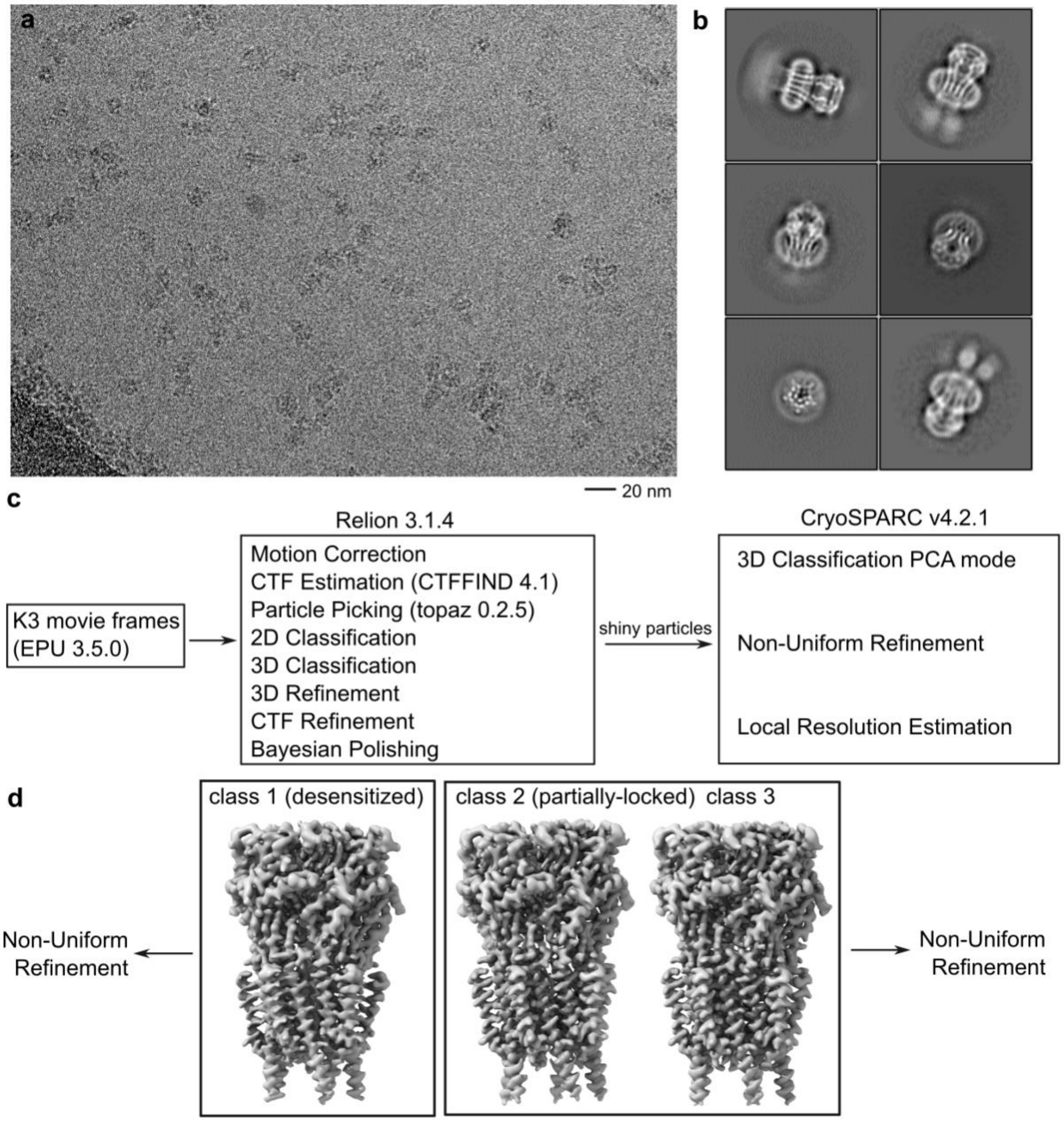

Supplementary Fig. 3. Processing pipelines for ρ1-EM structures.

(a) Representative cryo-EM image from the ρ1-EM with CGP36742 dataset.

(b) Representative 2D classification images from the ρ1-EM with CGP36742 dataset.

(c) Cryo-EM data processing workflow for the three datasets reported in this work.

(d) Representative 3D classification reconstructions from the ρ1-EM with GABOB dataset.

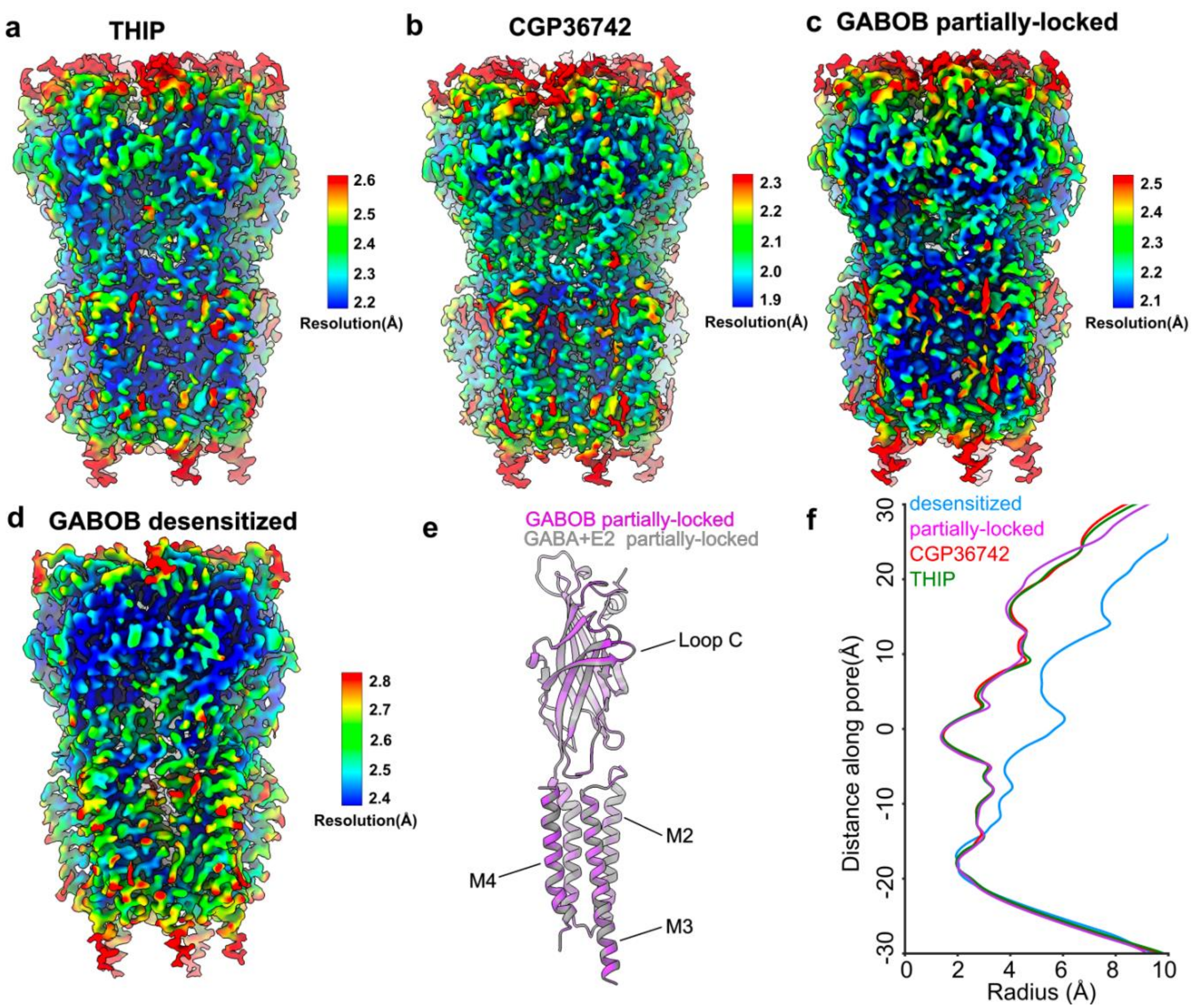

Supplementary Fig. 4. Local resolution and pore-radius profiles of the ρ1-EM structures.
(a-d) Cryo-EM maps colored by local resolution.
(e) Superimposed structures of ρ1-EM in the partially-locked state bound to GABOB (purple) and GABA+E2 (gray).
(f) Pore-radius profiles of ρ1-EM structures.

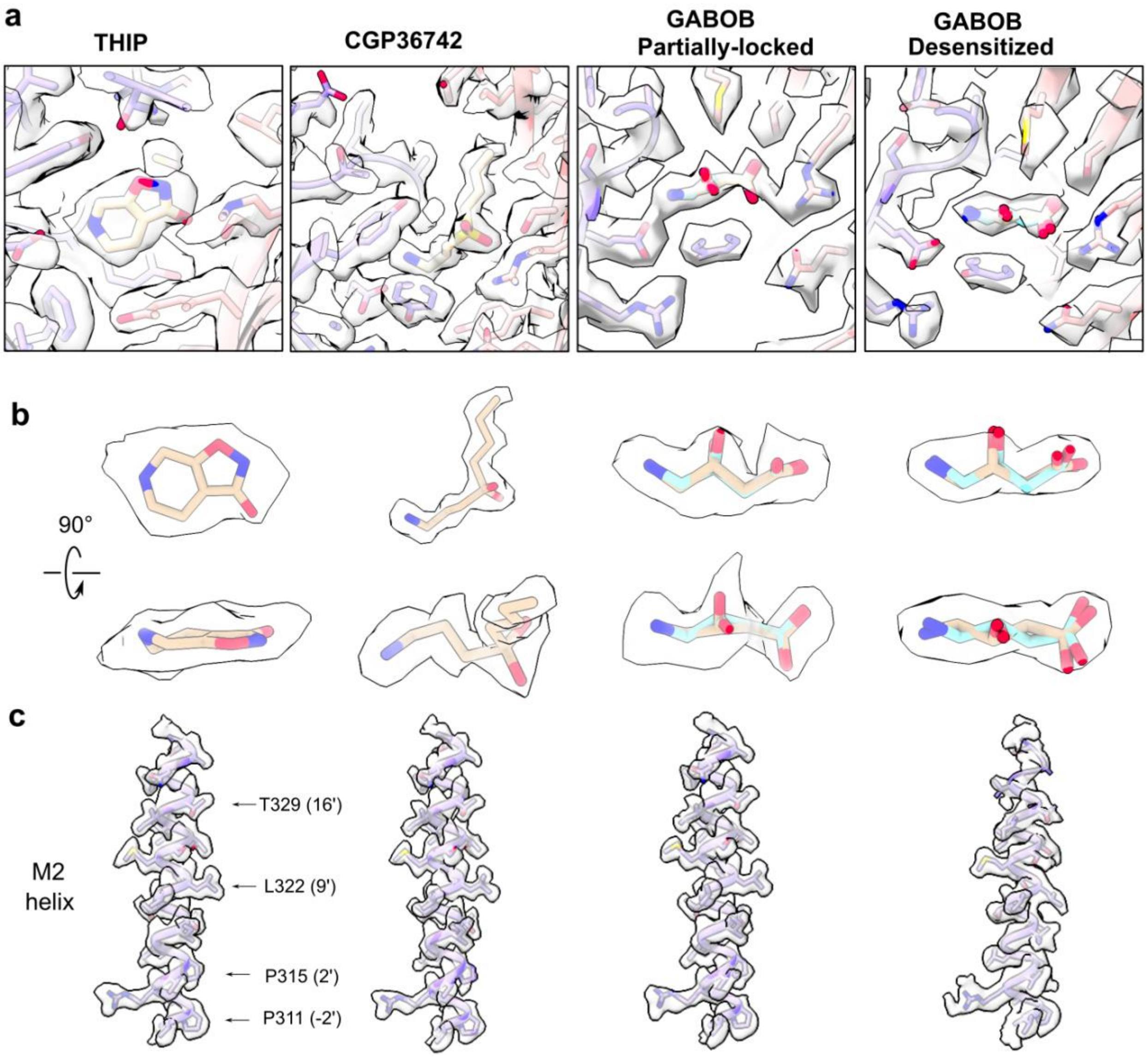

Supplementary Fig. 5. Representative densities of ρ1-EM structures.

(a) Densities and models of drug binding sites from the structures reported in this study.

(b) Densities and models of the ligands in two viewing angles.

(c) Densities and models of M2 helices from the structures reported in this study.

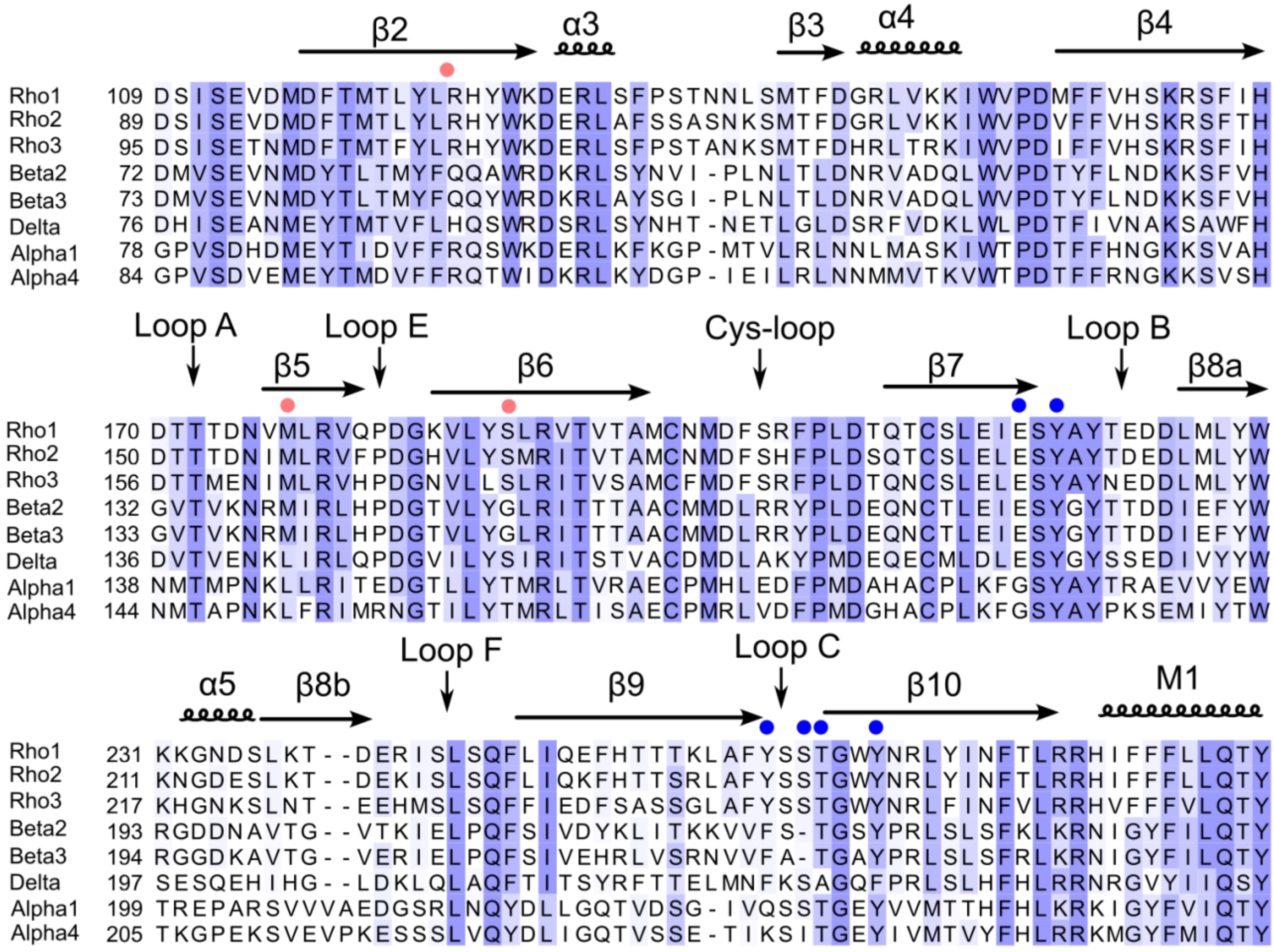

Supplementary Fig. 6. Sequence alignment of the orthosteric ligand binding region of representative human GABA$_A$ receptors. Residues are numbered according to reference UniProt sequences, with key structural features labeled above. Dots indicate positions involved in ligand binding from the principal (blue) and complementary (red) faces.

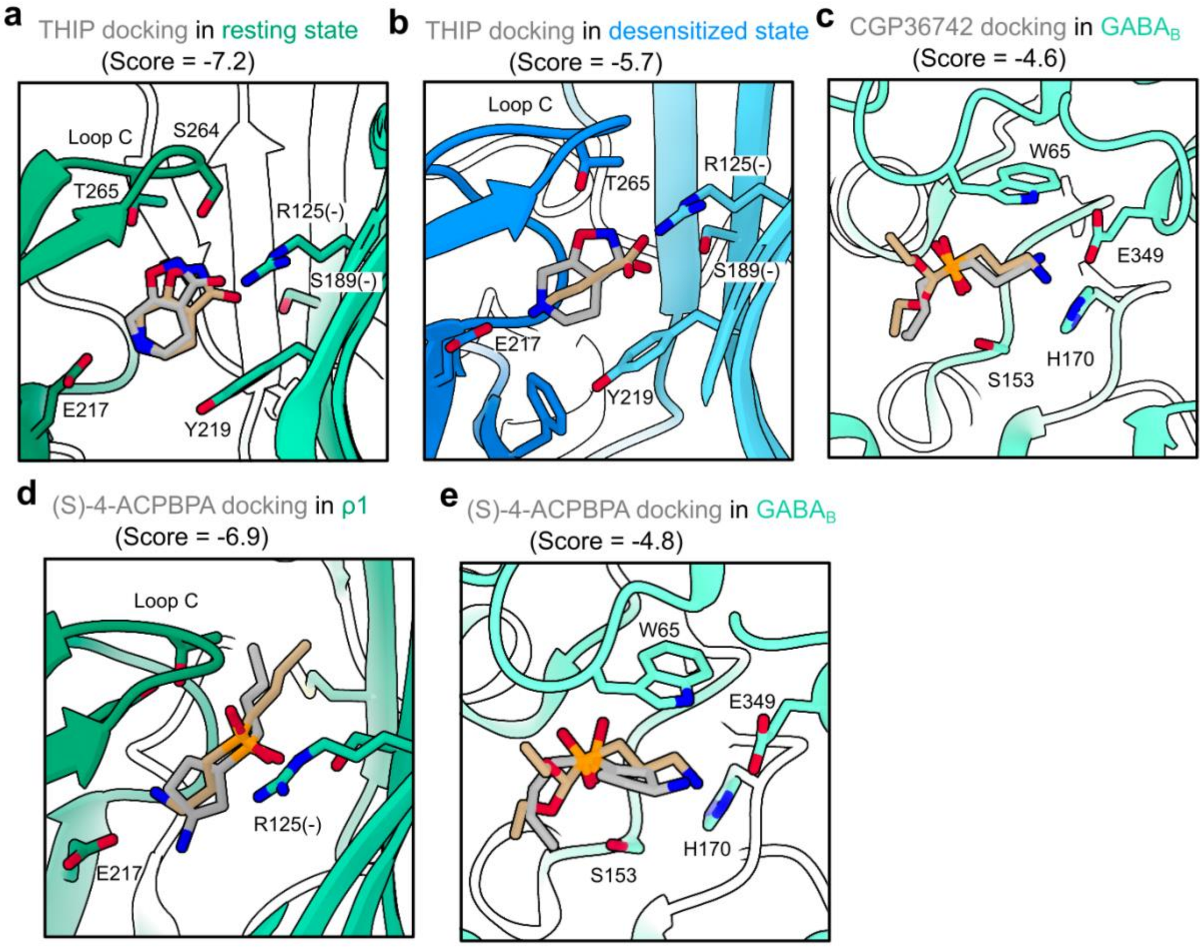

Supplementary Fig. 7. Computational docking of orthosteric ligands to ρ1-EM and $GABA_B$ receptor structures.

(a) Docking of THIP to ρ1-EM in the resting-like state (determined with THIP, green), with proximal residues of the orthosteric site shown as sticks. The best-scoring pose (gray, binding energy score above) is superimposed with THIP built manually into the cryo-EM density (tan). All sidechains and ligands are colored by heteroatom.

(b) Docking of THIP to ρ1-EM in a desensitized state (PDB ID 8RH8[27], blue), represented as in *a*. The best-scoring pose (gray, binding energy score above) is superimposed with GABA as built in the cryo-EM density (tan). The best docking pose for THIP in the desensitized state is less favorable than in the resting-like state.

(c) Docking of CGP36742 to a $GABA_B$ receptor (PDB ID 4MR8[38], green), represented as in *a*. The best-scoring pose (gray, binding energy score above) is superimposed with CGP35348 as built into the cryo-EM density (tan).

(d) Docking of (S)-4-ACPBPA to ρ1-EM in a resting-like state (green), represented as in *a*. The best-scoring pose (gray, binding energy score above) is superimposed with CGP36742 built manually into the cryo-EM density (tan).

(e) Docking of (S)-4-ACPBPA to a $GABA_B$ receptor (PDB ID 4MR8[38], green), represented as in *a*. The best-scoring pose (gray, binding energy score above) is superimposed with CGP35348 as built into the cryo-EM density (tan). The best docking pose for (S)-4-ACPBPA in the $GABA_B$ receptor is less favorable than in ρ1.

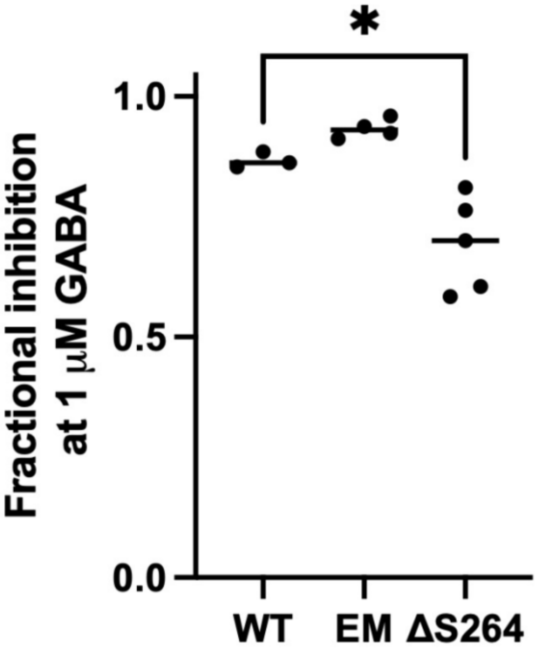

Supplementary Fig. 8. Electrophysiology profiles of ρ1 constructs. Comparison of inhibition of 1 μM GABA response by THIP for three ρ1 constructs. Asterisk indicates significance of $p < 0.05$ in a two-way *t*-test between wild-type and ΔS264 variants of full-length ρ1 ($p = 0.0151$).

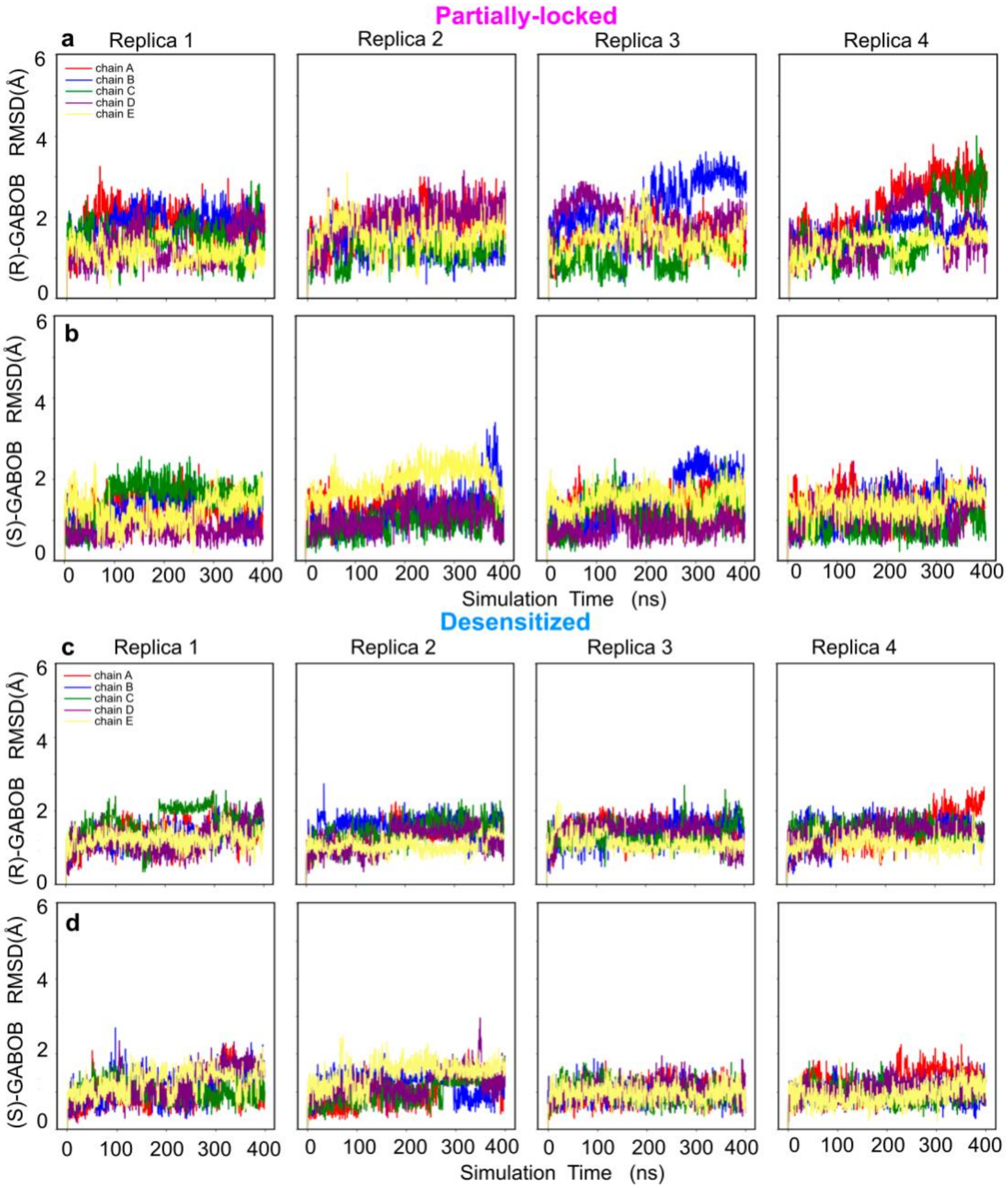

Supplementary Fig. 9. GABOB stability during MD simulations.

(a-b) Dynamics of (R)- (above) and (S)-GABOB (below) in the partially-locked state of ρ1-EM, calculated by RMSD and colored by chain.

(c-d) Dynamics of (R)- (above) and (S)-GABOB (below) in the desensitized state of ρ1-EM.

Supplementary Table 1. Molecular dynamics simulation parameters.

Apparent state	Partially-locked		Desensitized	
Ligand	(R)-GABOB	(S)-GABOB	(R)-GABOB	(S)-GABOB
Box dimensions (ÅxÅxÅ)	120 x 120 x 176	120 x 120 x 178	120 x 120 x 179	121 x 121 x 170
Number of atoms	264190	264269	267414	258679
Number of waters	60624	60651	61526	58619
Salt concentration	150 mM	150 mM	150 mM	150 mM
Number of lipids	455	455	455	455
